# A CRISPR-based approach for targeted DNA demethylation

**DOI:** 10.1038/celldisc.2016.9

**Published:** 2016-05-03

**Authors:** Xingxing Xu, Yonghui Tao, Xiaobo Gao, Lei Zhang, Xufang Li, Weiguo Zou, Kangcheng Ruan, Feng Wang, Guo-liang Xu, Ronggui Hu

**Affiliations:** 1 Key Laboratory of Systems Biology, CAS Center for Excellence in Molecular Cell Science, Innovation Center for Cell Signaling Network; Institute of Biochemistry and Cell Biology, Shanghai Institutes for Biological Sciences, Chinese Academy of Sciences, Shanghai, China; 2 University of Chinese Academy of Sciences, Shanghai, China; 3 State Key Laboratory of Cell Biology, Shanghai, China; 4 State Key Laboratory of Molecular Biology; Institute of Biochemistry and Cell Biology, Shanghai Institutes for Biological Sciences, Chinese Academy of Sciences, Shanghai, China; 5 Departments of Oral and Craniofacial Implant and Oral and Maxillofacial Surgery, School of Medicine, the Ninth People's Hospital Affiliated with Shanghai Jiao Tong University, Shanghai, China

**Keywords:** CRISPR, demethylation, RANKL

## Abstract

In mammalian cells, DNA methylation critically regulates gene expression and thus has pivotal roles in myriad of physiological and pathological processes. Here we report a novel method for targeted DNA demethylation using the widely used clustered regularly interspaced short palindromic repeat (CRISPR)-Cas system. Initially, modified single guide RNAs (sgRNAs) (sgRNA2.0) were constructed by inserting two copies of bacteriophage MS2 RNA elements into the conventional sgRNAs, which would facilitate the tethering of the Tet1 catalytic domain (Tet-CD), in fusion with dCas9 or MS2 coat proteins, to the targeted gene loci. Subsequently, such system was shown to significantly upregulate transcription of the target genes, including *RANKL*, *MAGEB2* or *MMP2*, which was in close correlation to DNA demethylation of their neighboring CpGs in the promoters. In addition, the dCas9/sgRNA2.0-directed demethylation system appeared to afford efficient demethylation of the target genes with tenuous off-target effects. Applications of this system would not only help us understand mechanistically how DNA methylation might regulate gene expression in specific contexts, but also enable control of gene expression and functionality with potential clinical benefits.

## Introduction

DNA methylation, an epigenetic process by addition of a methyl group to DNA, mainly occurs at the fifth carbon of cytosine base within CpG dinucleotide. In mammalian cells, DNA methylation regulates gene expression and thus has critical roles in myriad of physiological and pathological processes, which include, but are not limited to, cell development and differentiation, genome imprinting and tumorigenesis [1]. Typically, DNA hypermethylation in genes of tumor suppressors would silence their expression and contribute to multiple types of human cancers. On the other hand, DNA hypermethylation also etiologically underlies many devastating human neuromuscular diseases including fragile X-chromosome syndrome, in which an expansion of trinucleotide repeat (CGG) in the promoter region of *FMRP* led to hypermethylation of the gene and substantially suppressed the expression of the gene [2]. Therefore, advances in techniques for manipulation of the DNA methylation status of target genes will not only help us understand mechanistically how DNA methylation might regulate gene expression in a specific context, but enable controlling of gene expression and functionality with potentially beneficial clinical outcomes.

Recently, many enzymes have been found to catalyze active DNA demethylation with distinct mechanisms [3, 4]. Among them, Tet (ten eleven translocation) dioxygenase-catalyzed 5-methylcytosine oxidation was reported to promote DNA demethylation with Tet catalytic domain (Tet-CD) as the smallest functional module [5, 6]. Attempts in targeted DNA demethylation using transcription activator-like effector -fused TET1-CD have been tried to activate target genes [7]. However, its broader use has been restricted as the transcription activator-like effector-based strategies necessitate cumbersome design and assembly, thus being unsuitable for high-throughput applications.

Recently, bacterial clustered regularly interspaced short palindromic repeats (CRISPRs) system has been popularly applied into genome engineering and editing. With the deactivated endonuclease Cas9 (hereafter designated dCas9), versatile synthetic biology platforms have been developed to achieve gene regulation, genomic editing or fluorescent labeling [8]. Furthermore, a discovery of the unexpected plasticity in sgRNA has led to insertion of additional RNA elements to form the sgRNA2.0 system [9]. In principle, such RNA elements are recognized by some RNA-specific binding protein effectors, which would potentially lead to amplification in the efficacy of targeted dCas9-mediated functional moieties [9, 10].

Here, we demonstrate a strategy for targeted demethylation of specific genomic loci by tethering Tet1-CD both to MS2 RNA element-containing sgRNA2.0 system-guided dCas9 and MS2 bacteriophage coat protein [9]. Through efficient demethylation of the neighboring CpGs in specific gene loci, it was shown to upregulate the transcription of multiple genes, including *RANKL*, *MAGEB2* and *MMP2*, which strongly suggests a broad-future use of this system in manipulating DNA methylation in targeted manner.

## Results

### Design of CRISPR-Cas-based targeted DNA demethylation system

**Figure 1:**
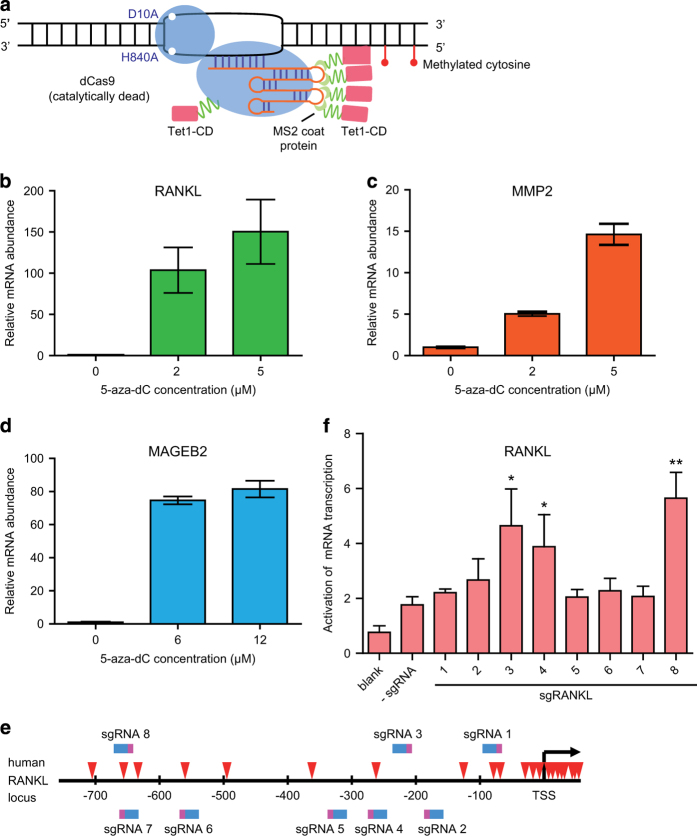
CRISPR-Cas-based targeted DNA demethylation system design. (**a**) Schematic description of targeted demethylation via sgRNA2.0-guided recruitment of dCas9- and MS2-fused Tet1-CD. (**b**–**d**) 5-Aza-dC treatment upregulated mRNA transcription of several genes as assayed by quantitative real-time PCR (qRT-PCR) in different cells: RANKL (**b**) and MMP2 (**c**) in HEK-293FT cells, and MAGEB2 (**d**) in HeLa cells. The respective 5-aza-dC concentration was labeled under the columns. (**e**) Eight sgRNAs were selected targeting regions within −800-bp upstream to the transcription start site (TSS) of human *RANKL* gene. The sgRNAs recognizing their respective target sites were shown in blue-pink color (pink color represented PAM region), with the CpG sites indicated with red arrowheads. (**f**) RANKL mRNA expression was assayed 4 days after co-transfection of sgRANKL-(1–8) guided dCas9- and MS2-Tet1-CD in HEK-293FT using qRT-PCR assay. Results were shown after normalization to the blank control (means±s.e.m., *n*=3). *P<0.05, **P<0.01 compared with -sgRNA group by unpaired *t*-test.

To construct the dCas9-mediated demethylation system, the D10A and H840A substitutions were introduced into Cas9 to form dCas9, which was subsequently fused to Tet1-CD using a flexible linker (Figure 1a and Supplementary Figure S1) to create dCas9-Tet1-CD. Meanwhile, U6 promoter-driven sgRNA2.0 transcription system was cloned into the same vector, with two MS2 RNA elements inserted into the sgRNA [9]. On the other hand, MS2 coat protein was fused to Tet1-CD with a flexible linker to form MS2-Tet1-CD. Guided by sgRNA2.0 targeting a specific sequence of the genome, both dCas9-Tet1-CD and MS2-Tet1-CD could be simultaneously recruited to the target gene locus.

Expression of human *RANKL*, *MMP2* and *MAGEB2* genes has been previously reported to be repressed because of DNA hypermethylation in HEK-293 cells [11, 12]. As shown in Figure 1b-d, the expression of all these three genes was found to be significantly upregulated in HEK-293FT and HeLa cells upon treatment with 5-aza-2′deoxycytidine, a potent inhibitor of DNA methyltransferase, (2–12 μM, respectively, 4 days). Therefore, DNA hypermethylation in these genes might have indeed repressed their expression, which could potentially be reversed by targeted demethylation of the genomic loci.

To explore this possibility, eight sgRNAs were designed to target the promoter region (–800-bp upstream of the transcription start site) of human *RANKL* gene and their individual impact on the transcription of *RANKL* mRNA was examined (Figure 1e). After transfection of HEK-293FT cells with dCas9-Tet1-CD and MS2-Tet1-CD (0.14 pmol and 0.36 pmol per 1×10^6^ cells, respectively) for 4 days, several sgRNAs (3, 4 and 8) were found to increase transcription of *RANKL* mRNA more significantly than the others (Figure 1f). Among them, sgRANKL-3 and 8 (designated as R3 and R8) were selected for further applications.

### Upregulation of target *RANKL* gene showed specific DNA demethylation-dependent effects

**Figure 2:**
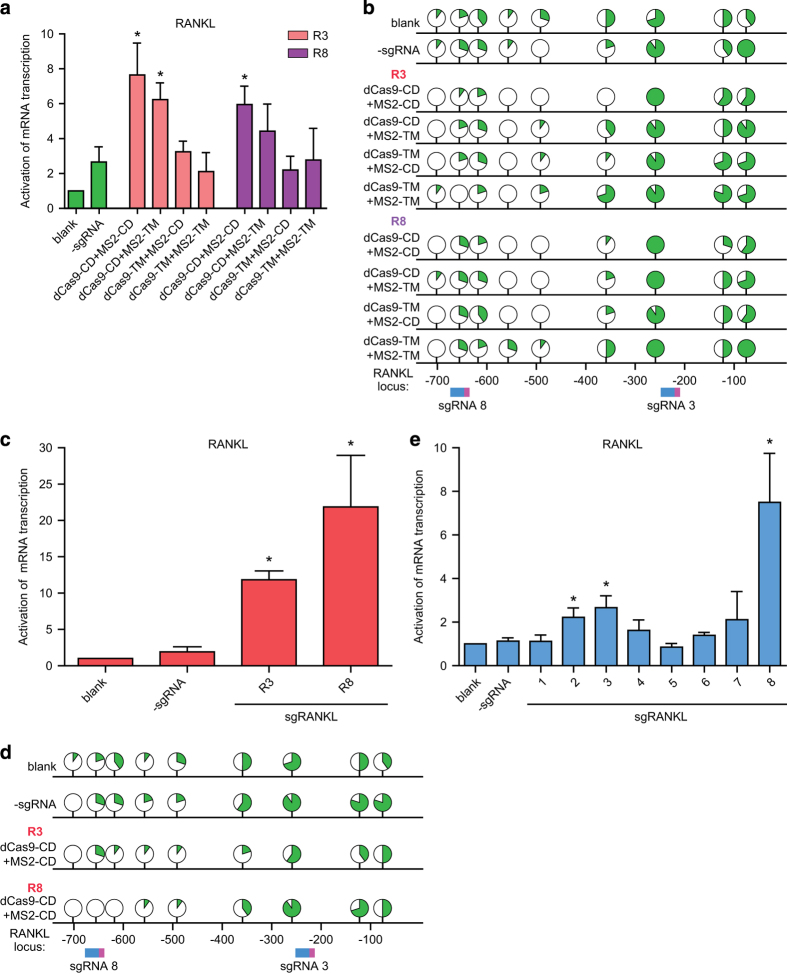
*RANKL* gene upregulation showed DNA demethylation-dependent manner. (**a**) Quantitative real-time PCR (qRT-PCR) assay was performed to assess the abundances of RANKL mRNA in HEK-293FT cells in indicated groups. (**b**) The percentages of methylated or unmethylated DNA at each site in *RANKL* gene, determined by bisulfite sequencing, were represented by green or white sections in the filled circles, respectively, with the numeric information listed in Supplementary Table S2. The sgRNAs recognizing their respective target sites were shown in blue-pink color (as in Figure 1e). (**c**) mRNA levels of RANKL were examined in HEK-293FT cell that stably expressed the demethylation system using qRT-PCR assay. (**d**) In the HEK-293FT cells stably expressing the demethylation system, the percentages of methylated or unmethylated DNA at each site in *RANKL* gene were determined through bisulfite sequencing, respectively. The corresponding detailed numeric data were listed in Supplementary Table S3. The sgRNAs recognizing their respective target sites were shown in blue-pink color (as in Figure 1e). (**e**) RANKL mRNA expression was assayed 4 days after co-transfection of sgRANKL-(1-8) guided dCas9- and MS2-Tet1-CD in SH-SY5Y cell line using qRT-PCR assay. Data were shown after normalization to the controls (blank group) (means±s.e.m., *n*=3). *P<0.05, compared with -sgRNA group by unpaired *t-*test.

In order to determine whether upregulation of *RANKL* gene expression indeed originated from locus-specific DNA demethylation activity of Tet1-CD, mutations of H1652Y/D1654A were introduced to create catalytically dead form of Tet1-CD in the dCas9 or MS2 fusion, resulting in dCas9-Tet1-TM or MS2-Tet1-TM, respectively [13]. As shown in Figure 2a, transcription of *RANKL* mRNA was found to have diminished in cells expressing dCas9-Tet1-TM, MS2-Tet1-TM or both, indicating that dioxygenase activities were required for the dCas9/sgRNA2.0-induced gene activation. Specifically, when applied with R3 and R8, the enzymatically dead dCas9-Tet1-TM totally lost its activating effect on the transcription of *RANKL* mRNA, further underscoring the requirement of dioxygenase activity for dCas9-Tet1-CD-mediated activation of RANKL mRNA transcription. Meanwhile, when the dioxygenase activity of dCas9-fused Tet1-CD was intact, a simultaneous application of MS2-Tet1-CD led to approximately 1.5-fold increase in activation of *RANKL* gene transcription (Figure 2a). Certainly, for comparison, dCas9-Tet1-CD, MS2-Tet1-CD or their catalytically dead mutants were expressed in these cells at comparable levels to exclude the possibility that the above observed differences might be caused by variations in expression of the fusion proteins (data not shown).

In order to further check whether the upregulated transcription of *RANKL* gene was a direct result of targeted demethylation that occurred at the specific *RANKL* gene sequence, the methylation status of sgRANKL-targeted loci was examined using bisulfite-sequencing approach [14]. As shown in Figure 2b (also see Supplementary Table S2), co-expression of both dCas9-Tet1-CD and MS2-Tet1-CD did lead to removal of the methyl groups in the neighboring CpGs, which was closely correlated to the increase in mRNA transcription of *RANKL* gene in individual groups.

In addition, in cells stably expressing the demethylation system including dCas9-Tet1-CD and MS2-Tet1-CD, the transcriptional levels of *RANKL* were prominently elevated in the R3 (~12 folds) or R8 group (~22 folds; Figure 2c), when compared with that of the cells that transiently expressed the demethylation system (Figure 2a). Meanwhile, the methyl groups at the R3 or R8 sgRNA target sites were more effectively removed in the stable expression groups, suggesting that a very likely causing effect of the site-specific demethylation events (Figure 2d and Supplementary Table S3). Altogether, we have shown that dCas9-based demethylation could indeed upregulate mRNA transcription through sequence-specific demethylation of the target gene.

To further explore the general applicability of our dCas9-based demethylation approach, we tested the demethylation efficiency of the sgRNA2.0-guided system in SH-SY5Y cells, a human neuroblastoma cell line. As shown in Figure 2e, three out of all eight sgRNAs tested so far demonstrated significant activation of *RANKL* gene transcription. Among them, the dCas9 system with R8 manifested the highest efficiency in upregulating mRNA transcription of the target gene (increased six folds) (Figure 2e). Therefore, the efficacy of this dCas9-based system seemed not confined to only a specific cell type.

### Optimization of the efficacy of dCas9-based system and exhibition of functional consequences

**Figure 3:**
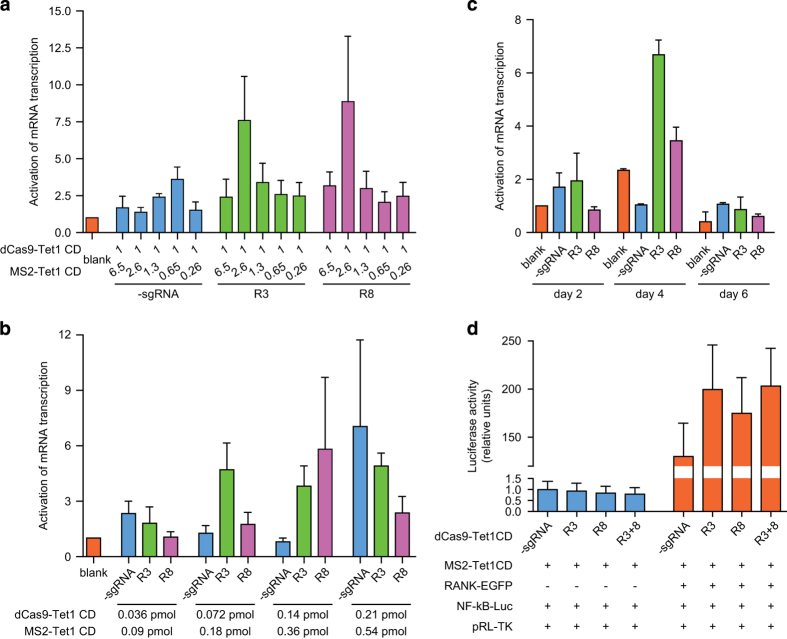
Optimization of dCas9-based demethylation system. (**a**) Quantitative real-time PCR (qRT-PCR) data showed *RANKL* gene expression changes after transfection of different molar ratios of dCas9-Tet1-CD and MS2-Tet1-CD at equal total moles in HEK-293FT cells. *X* axis, molar ratios of transfected components individually, in total amount of 0.5 pmol per well in a 12-well plate format. (**b**) qRT-PCR data examined the changes in RANKL mRNA after transfection of different amounts of the system components to eliminate random demethylation effects in HEK-293FT cell line. *X* axis, listed molar quantities of transfected components (pmol/well in the format of 12-well plate). (**c**) Assessment of mRNA transcription of *RANKL* gene along the time course after transfection of the dCas9-based demethylation system (days 2–8) in HEK-293FT cells. (**d**) Dual luciferase-based NF-κB reporter assays were performed in HEK-293FT cells expressing exogenous RANK or not. They were co-transfected with sgRNA2.0-guided demethylation, with the luciferase activities (in relative units) normalized to that of the -sgRNA group. Blank, cells transfected with the expression vectors only; -sgRNA, cells co-transfected with dCas9- and MS2-Tet1-CD but not any sgRNAs targeting RANKL sites; TM, Tet1-CD with H1652Y/D1654A mutations. Data were shown after normalization to the controls (blank group) (means±s.e.m., *n*=3).

To further optimize the efficacy of this dCas9-based system, combinations of different ratios and dosages of dCas9-Tet1-CD and MS2-Tet1-CD were titrated (Figure 3a). As shown in Figure 3a, both R3 and R8 sgRNA groups upregulated the transcription of *RANKL* gene with a high efficiency (seven- to eight folds) at the molar ratio of 1:2.6 (dCas9-Tet1-CD: MS2-Tet1-CD), when compared with control groups.

In order to minimize the potential random demethylation of human genome caused by forced expression of dCas9-Tet1-CD and MS2-Tet1-CD, the demethylation efficiencies by different total amounts of these two components with a fixed molar ratio (1:2.6) were investigated. As shown in Figure 3b, when normalized to blank group, 0.5 pmol of total amount (dCas9-Tet1-CD: MS2-Tet1-CD, 0.14 pmol: 0.36 pmol/1×10^6^ cells) gave the robust activation (three- to five folds) for *RANKL* gene expression in combination with either R3 or R8 sgRNAs, whereas the control group containing no sgRNA exhibited little or no effect.

Next, we sought to examine the demethylation efficiency of this sgRNA2.0-guided system along the time course after its introduction into the cells, by assessing the mRNA expression levels for *RANKL* gene at different days (days 2–6) post-transfection (Figure 3c). As shown in Figure 3c, when R3 sgRNA was used, the maximal activation of *RANKL* gene expression (>6 folds, compared with no-sgRNA group) was achieved at day 4 post transfection, and dropped significantly at other time points (days 2–6). Expectedly, with R8 as the sgRNA, the demethylation efficiency of the dCas9-based system fluctuates in a similar time-dependent pattern (with a maximal increase of *RANKL* gene expression at 2.5 folds at day 4).

Conceivably, the above observed maximal demethylation efficacy at day 4 post transfection in the current settings might result from the time taken for the cells to express high molecular weight protein components (dCas9-Tet1-CD, etc) at decent levels, and that to assemble the functional entity for active demethylation. Therefore, the time for the dCas9-based system to manifest its best DNA demethylation efficacy in a specific setting may be determined by multiple factors, such as different target genes, individual combinations of dCas9-sgRNAs and the types of host cells for such operation, which should be assessed contingently.

Critically, we next asked whether demethylation of *RANKL* gene by the dCas9/sgRNA2.0-guided system could indeed functionally impact the intracellular *RANK*/NF-κB signaling pathway. In a typical NF-κB luciferase reporter assay [15], as shown in Figure 3d, activity of the *RANK*/NF-κB signaling pathway was at basal level in human embryonic kidney 293FT cells that expressed low level of *RANKL* mRNA because of hypermethylation in *RANKL* gene. It also expressed endogenous *RANK* protein at almost undetectable level [15]. Only upon re-introduction of *RANK* and upregulation of *RANKL* gene expression through target demethylation, could the functional *RANKL*/NF-κB signaling pathway be re-instituted in these cells. Indeed, as shown in Figure 3d, the presence of *RANK* protein appeared to be a pre-requisite for the *RANKL*-activated NF-κB signaling pathway, as the cells expressing no *RANK* protein gave little or no signal in NF-κB signaling reporter assays, in disregard of introduction of the dCas9 system. Meanwhile, in cells stably expressing *RANK*, transfection of R3, R8 or both manifested marked increase in activation of NF-κB signaling, when compared with no-sgRNA control group. Altogether, these data clearly demonstrated that targeted re-writing of the methylation status of *RANKL* gene by this dCas9-based demethylation system could indeed efficiently manipulate gene expression, with obvious functional consequences.

### Evaluation of off-target effects of dCas9-based demethylation system

Evidently, the off-target effect has constituted a major concern in applications of any Cas9-based technique [16–18], and our dCas9-based demethylation would not be an exception. At present, off-target recognition by dCas9 was attributed to the following factors: the sequence similarities between PAM regions used in dCas9, the numbers of mismatches in the off-target loci compared with those of the target loci, the distance of mismatches to the PAM region, and the homeostatic levels of sgRNAs and Cas9 protein in host cells.

Taken these into consideration, efforts have been taken in every step of the current approach to minimize potential off-target effect of the dCas9-based demethylation system. These included: (1) using web-based scoring algorithm [16] to help design sgRNAs that precisely targeted the genome loci of interest; (2) carefully titrating the amounts and ratios of different components in our system (see above Figure 3a and b). In addition, it was found that dCas9 could also potentially interfere with the mRNA transcription of the susceptible genes mainly at initiation and elongation steps [19].

**Figure 4:**
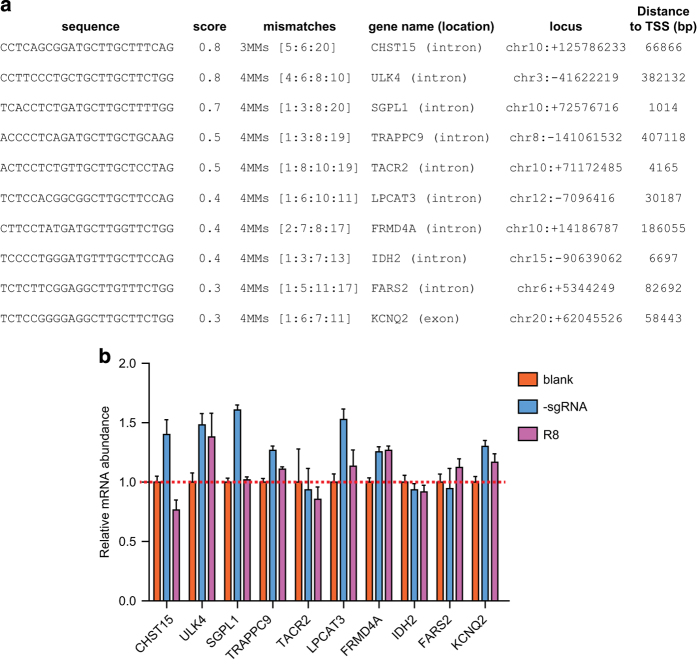
Assessment of the off-target effect of the dCas9-based system. (**a**) The top 10 potential off-target sites predicted for sgRANKL-8 (R8) were selected using a scoring system described previously [16]. Sequences in the first column showed DNA sequences of the potential off-target sites; the numbers of mismatches indicated the numbers of basepairs (bp) in off-target sites that were different from those in the authentic R8-targeting site. The bp numbers indicated the locations of off-target sites in related chromosomes. (**b**) Changes in expression levels of RANKL mRNA in cells co-transfected with R8 sgRNA and dCas9- and MS2-Tet1-CD, compared with those of the blank or sgRNA controls. Quantitative real-time PCR (qRT-PCR) assays were performed with cells of the indicated groups. Sequences of the specified primers were listed in Supplementary Table S5. Data were shown after normalization to the controls (blank group) (means±s.e.m., *n*=3).

Therefore, we chose the top 10 potential off-target gene loci according to the scoring values, and examined whether their mRNA transcription levels were altered as a result of potential interference from dCas9 or random demethylation mediated by Tet1-CD moiety (Figure 4a). As shown in Figure 4b, no significant changes in the mRNA expression levels for these potential off-target genes have been detected between those in the group transfected with R8 sgRNA and the control groups, in which no dCas9-Tet1-CD or sgRNA was applied. Therefore, dCas9/sgRNA2.0-directed demethylation system is seemingly capable of efficient demethylation of target gene with tenuous off-target effects.

### Wide-range gene targeted demethylation effects implemented by dCas9-based system

Finally, to determine whether this dCas9-based demethylation system would function in wide-range of gene contexts, its efficacy was also tested on other genes (MAGEB2 and MMP2) whose expression seemed to be also repressed by DNA hypermethylation (Figure 1c and d).

**Figure 5:**
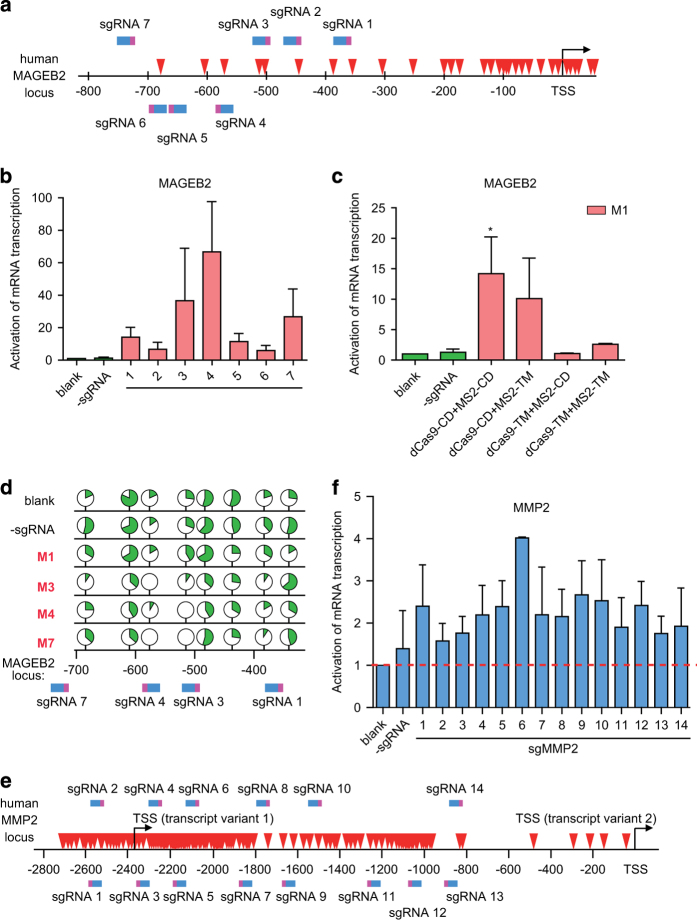
Generality of gene targeted demethylation of the dCas9-based system. (**a**) Seven sgRNAs were designed to target the promoter region (−800 to −300-bp upstream of the transcription start site) of human MAGEB2 gene. (**b**) Quantitative real-time PCR (qRT-PCR) was carried out to assess the abundances of MAGEB2 mRNA in indicated groups. (**c**) Changes in the mRNA transcription of MAGEB2 gene were evaluated upon co-transfection of sgRNA (M1) with combinations of the indicated chimeric Tet1-CD or their respective mutants in HeLa cells. *P<0.05, compared with -sgRNA group by unpaired *t*-test. (**d**) The methylation states of DNA in the promoter region of MAGEB2 gene in each group were examined using bisulfite-sequencing approach and presented as above, with the detailed information listed in Supplementary Table S3. The sgRNAs recognizing their respective target sites were shown in blue-pink color (as in **a**). (**e**) Fourteen sgRNAs were designed to target sequences within the promoter region (−2 800-bp upstream of the transcription start site) of human MMP2 gene. Two transcript variants with different TSSs were shown here. (**f**) Activation of MMP2 mRNA transcription by sgMMP2-guided dCas9 demethylation system in HEK-293FT cells. The red dash line was used for easy comparison of the activation of mRNA transcription between the blank group and those of different groups. Data were shown after normalization to the controls (blank group) (means±s.e.m., *n*=3).

A set of seven sgRNAs were thus designed to target regions located from −800 to −300-bp upstream to the transcription start site of *MAGEB2* gene (Figure 5a). Among the selected sgRNAs, co-transfection of dCas9-Tet1-CD and MS2-Tet1-CD (0.14 pmol and 0.36 pmol/1×10^6^ cells, respectively) in HeLa cells for 4 days led to 5- to 60-fold increase in the mRNA transcription of *MAGEB2*, compared with that for control group (Figure 5b). As shown in Figure 2a, Tet1-CD activity seemed also required for the dCas9-based demethylation system using sgMAGEB2-7 (M7) (Figure 5c). Apparently, the methylation status of the target region was inversely correlated to that of mRNA transcription of *MAGEB2* gene (Figure 5d and Supplementary Table S4).

In another attempt with 14 sgRNAs designed to target the CpG-dense islands in the promoter of *MMP2* gene (Figure 5e), one to threefold increase in mRNA transcription of the *MMP2* gene was typically observed in cells expressing the dCas9-based system together with specific sgRNA (Figure 5f).

Therefore, taking into account of the demethylation efficiencies of the dCas9 system targeting *RANKL*, *MAGEB2* and *MMP2* genes, one might safely conclude the current system has enabled targeted demethylation and activation of gene expression independent of specific gene contexts.

### The non-additive effect of the dCas9-based demethylation system

**Figure 6:**
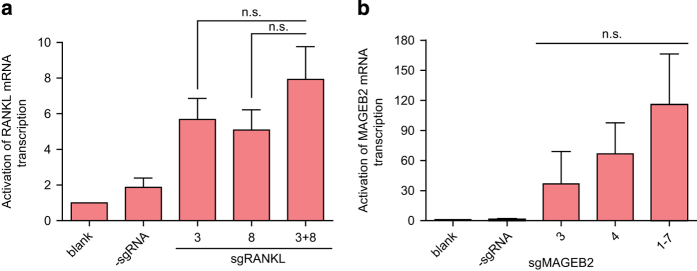
The non-additive effect of upregulating gene expression in dCas9-based demethylation system. (**a**) The non-additive effect of R3 and R8 sgRNAs-guided demethylation in activation of mRNA transcription of human *RANKL* gene in HEK-293FT cells, in comparison with that of single sgRNA (R3 or R8). (**b**) The non-additive effect of M1-7 in comparison with prominent single sgRNAs (the same results showed in Figure 5b) in HeLa cells. n.s., not significant from unpaired *t*-test. Data were shown after normalization to the controls (blank group) (means±s.e.m., *n*=3).

So far, we have demonstrated that the dCas9/sgRNA2.0-based demethylation system could be individually applied to demethylate single genes and activate their expression (Figures 1f, 2c and e, 5b and f). As shown in Figure 6a and b, this dCas9/sgRNA2.0-based demethylation system can also be used simultaneously to manipulate DNA methylation and gene expression of both *RANKL* and *MAGEB2* genes.

Interestingly, when the sgRNAs targeting different sites in *RANKL* and *MAGEB2* were introduced into the cells simultaneously, no additive or synergistic effects were observed in upregulating the transcription of the respective target genes (Figure 6a and b). This finding was highly reminiscent of a previous report in which dCas9-fused p300 histone acetyltransferase core domain was used to activate gene expression individually [20]. Meanwhile, such non-additive effect was clearly in sharp contrast to the synergistic effect previously reported for the CRISPR-guided VP64 system [21, 22], which might result from the difference between the VP64-recruited multiple transcriptional initiation complex [23] and the Tet1-CD catalyzed individual demethylation reaction.

## Discussion

In summary, we have sufficiently demonstrated that this dCas9-based system, additionally empowered by sgRNA2.0-guided target recognition and MS2-Tet1-CD, could precisely demethylate specific regions of target genes and achieve substantial activation of gene expression.

Interestingly, when sgRANKL-3 (R3) was used for DNA demethylation of *RANKL* gene, the methylation status of the CpGs region near the targeted sites was not altered, but those for the CpGs region (100–300-bp distance) instead were significantly removed (Figure 2b). Similar effect was also observed when sgMAGEB2-7 (M7) was used to demethylate *MAGEB2* gene (Figure 5d). Currently, we believe there might be a couple of underlying factors functioning independently or concertedly to contribute to this repeatedly observed phenomenon. For example, owing to steric hindrance, some sgRNA-targeted sites or their nearby sequences might be hard to access by dCas9-guided system components. Moreover, the methylation status of a given sequence might be determined by the balance between the functionality of both endogenous methylation/demethylation machineries and the introduced dCas9-based demethylation system.

Remarkably, upregulating expression of endogenous genes represented an obvious advantage, given the fact that 95% human genes encode alternatively spliced transcript [24] and forced expression of any single mRNA transcript would distort the original homeostatic ratios of the endogenous transcripts. In addition to the existing techniques using zinc-finger protein or transcription activator-like effector [7, 25, 26], our dCas9-based demethylation system is supplementing the current toolbox with an outstanding capacity for locus-specific epigenetic editing.

An ongoing effort of our future study is to apply this dCas9-based demethylation system in animal models for human diseases etiologically caused by gene hypermethylation. Given the ease and simplicity in programming its specificity, this dCas9-based demethylation system may also find use in manipulating multiplex demethylation in genome-wide scale. Conceivably, histone modifiers or chromatin remodeling factors could be applied in lieu of or together with Tet1-CD dioxygenase to achieve specific effects in epigenetic editing [20, 25, 27] or manipulation of the conformation of a fragment of chromatin for research or clinical use.

As shown in Figures 1f, 2e and 5b it was also notable that most of the high-performance sgRNAs (R3 or R8 targeting *RANKL,* or M1, M3 and M7 for *MAGEB2*) seemed to pair up with the antisense strands of the target genes. Potentially, in this configuration, the pre-specified order of the sgRNA and MS2 sequences in the sgRNA2.0 synthetic module might favor the recognition of the targeted sites by the dCas9-Tet1 CD and MS2 coat protein fused Tet1-CD, which may consequently facilitate demethylation of the targeted region and promote gene transcription. Certainly, it remains to be further investigated whether the sense strand-derived sgRNAs could constantly lead to generation of high-performance demethylation.

In previous reports, VP64 effector domains exerted transactivation effects by recruiting several transcription co-factors [9], whereas p300 histone acetyltransferase core domain by catalyzing chromatin histones at gene enhancers [20]. In our current system, by manipulating DNA methylation and demethylation status of specific sites, it would allow us to mechanistically explore how specific methylated or non-methylated CpGs might affect gene expression levels and neighboring chromatin states, further functionally impacting cell physiology and pathology.

Therefore, applications of the RNA-guided dCas9 demethylation techniques should have versatile and broad applications in both basic research and medical therapeutics.

## Materials and Methods

### Plasmids construction

The pdCas9-Tet1-CD plasmid was constructed based on parental plasmid pX330, which harbored two expression cassettes (a kind gift by Dr Feng Zhang, MIT, USA). Cas9 was also subject to D10A/H840A substitution to construct dCas9, and MS2 RNA elements were inserted into U6 promoter-driven sgRNA cassette at two different sites according to the previous report (briefly designated as sgRNA2.0) [9]. Mouse Tet1-CD was fused to dCas9 domain with a flexible linker (detailed sequences shown below) using Gibson assembly method [28]. MS2 coat protein gene fused with Tet1-CD was inserted into pcDNA3.1/hygro(+) vector (Life Technologies, Carlsbad, CA, USA) using Gibson assembly method. The full amino-acid sequences of dCas9-Tet1 CD and MS2-NLS-Tet1 CD protein were provided in Supplementary Methods. Designed sgRNAs (listed in Supplementary Table S1) were synthesized as oligos (Sangon, Shanghai, China), annealed and cloned into pdCas9-Tet1-CD vector at *Bbs*I digestion sites.

### Cell culture and transfection

HEK-293FT (Life Technologies), SH-SY5Y (ATCC, Mannassas, VA, USA) and HeLa (ATCC) cell lines were all cultured in Dulbecco’s modified Eagle’s medium (Corning, Corning, NY, USA) supplemented with 10% fetal bovine serum (Biochrom, Berlin, Germany) and penicillin/streptomycin (Life Technologies). Cells were incubated at 37 °C in a humidified 5% CO_2_ air incubator.

HEK-293FT and HeLa cell lines were treated with 5-aza-2′ deoxycytidine (Sigma, St Louis, MO, USA) at different dosages for 4 days, densities of the starting cell were approximately 12 500 cells per cm^2^; culture medium was changed every 2 days with new drug administration. Total RNAs were harvested 4 days post treatment.

In demethylation experiments, cells were seeded in 12-well format (Corning) and both pdCas9-Tet1-CD and pcDNA3.1-MS2-Tet1-CD plasmids were transfected into HEK-293FT using polyethylenimine (Sigma) and SH-SY5Y and HeLa cell lines using Lipofectamine 2000 (Life Technologies), respectively, according to the manufacturer’s protocols. Cells were harvested 4 days after transfection. Although in time course experiments, cells were harvested every 2 days post transfection until day 6.

To establish the cell lines that stably expressed the demethylation system, HEK-293FT cells were initially transfected with pcDNA3.1-MS2-Tet1-CD plasmid and subjected to sustained hygromycin treatment (75 μg ml^−1^, medium change per 3 days). The MS2-Tet1-CD-expressing cells were then transfected with pdCas9-Tet1-CD plasmids, and subjected to screening for hygromycin (75 μg ml^−1^) and puromycin (1 μg ml^−1^, medium change per 3 days) resistance to obtain cells that stably expressed both MS2-Tet-CD and dCas9-Tet1-CD. Colonies were picked and harvested for RNA and genomic DNA extraction to examine the expression and DNA demethylation of the targeted genes.

### NF-κB luciferase reporter assay

HEK-293FT cells were seeded in 12-well format and both pdCas9-Tet1-CD and pcDNA3.1-MS2-Tet1-CD plasmids were transfected using polyethylenimine reagents. Two days later, cells were seeded into 12-well plates. At day 3, pHAGE-RANK-EGFP, pGL3-NF-κB and pRL-TK plasmids were co-transfected using polyethylenimine reagents. Twenty-four hours later, dual-luciferase activity was measured following the manufacturer’s instruction (Promega, Madison, WI, USA).

### Quantitative real-time PCR

Total RNAs were extracted from cells of indicated groups using RNAsimple total RNA kit (Tiangen, Beijing, China). Complementary DNA was synthesized using ReverTra Ace qPCR RT Master mix (Toyobo, Osaka, Japan). Quantitative real-time PCR assay was performed to assess the relative abundances of the mRNAs of interest, using specific primers of sequences listed in Supplementary Table S5, stained SYBR Green (Toyobo) on CFX96 real-time PCR system (Bio-Rad, Hercules, CA, USA) to examine gene expression. The relative abundances of the transcripts of indicated genes were normalized to that of *GAPDH* gene, using the ΔΔCt method [29]. All data were obtained from at least three independent experiments.

### Bisulfite DNA sequencing

Genomic DNA was extracted from cells of indicated groups using the standard phenol–chloroform extraction method. Genomic DNA was treated with bisulfite using CpGenome Turbo Bisulfite Modification Kit (Millipore, Billerica, MA, USA) according to the manufacturer’s manual. The modified DNA was amplified using Platinum Taq DNA polymerase (Life Technologies) with the respective primer sets that recognize bisulfite-modified DNA only (primer sequences listed in Supplementary Table S6). Then the PCR products were cloned into pMD 18-T vector (Takara, Shiga, Japan), followed by Sanger sequencing.

### Statistics

Statistical analyses were performed with unpaired Student’s *t*-test using GraphPad Prism (version 5 for Windows, GraphPad Software, San Diego, CA, USA).
