# Supplementary material for: A CRISPR-based approach for targeted DNA demethylation
Source: Cell Discov. 2016 May 3;2:16009–. doi: 10.1038/celldisc.2016.9 (PMC4853773; doi:10.1038/celldisc.2016.9)
Supplement: Supplementary Figure S1 [file celldisc20169-s1.pdf]

Supplementary Figure S1

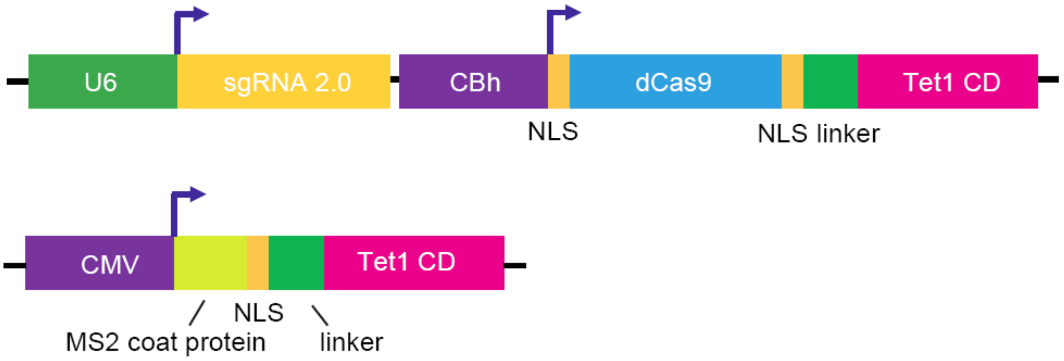

Supplementary Figure 1

Schematic representation of sgRNA2.0-guided demethylation system expression cassettes. The U6-driven sgRNA2.0 scaffold and hybrid CBA promoter (CBh)-driven dCas9-Tet1-CD cassette were on the same plasmid. On the backbone of another vector, the expression of MS2 coat protein-fused Tet1-CD (MS2-Tet1-CD) was driven by CMV promoter. Detailed amino acid sequences were displayed in Supplementary Materials I and II.
