# Supplementary material for: A CRISPR-based approach for targeted DNA demethylation
Source: Cell Discov. 2016 May 3;2:16009–. doi: 10.1038/celldisc.2016.9 (PMC4853773; doi:10.1038/celldisc.2016.9)
Supplement: Supplementary Table S1 [file celldisc20169-s2.pdf]

Supplementary Table 1 Sequences of sgRNA target sites used in the construction of sgRNA2.0-expression plasmids.

**sgRNA**	**Target site sequence (PAM region)** Red color highlighted PAM region, which should be omitted in sgRNA oligo synthesis.
sgRANKL-1	GTGGAAGAGGCAGCCTCGCC**TGG**
sgRANKL-2	TCCTTCATGGGGGCCCTCCA**AGG**
sgRANKL-3	CAAGGGGAGTCTGGAACCAC**TGG**
sgRANKL-4	ATGGGTTAAAGAAGACGCAG**AGG**
sgRANKL-5	CTCCTGCATCCATTCTAGTT**TGG**
sgRANKL-6	CTTGAAGGTGACATTGAGCG**AGG**
sgRANKL-7	TAGCCAGAAGCAAGCATCCG**AGG**
sgRANKL-8	CCTCCTCGGATGCTTGCTTC**TGG**
sgMAGEB2-1	CTTGGCTTTCACGGGAATCA**AGG**
sgMAGEB2-2	CACCTGTATAGTCTCGGGGA**AGG**
sgMAGEB2-3	TCGCCATTGTTAGCACCGAG**AGG**
sgMAGEB2-4	AGAGCCCCCTCGTAACACTT**AGG**
sgMAGEB2-5	GGGCCATTTCCACTAGTCCA**AGG**
sgMAGEB2-6	AACACACGAGGCATATTGAT**GGG**
sgMAGEB2-7	CACCAATGCTGTCACCCTTG**GGG**
sgMMP2-1	ACTTGCCTCTCTCGCGATCT**GGG**
sgMMP2-2	GGTGACGAGGTCGTGCACTG**AGG**
sgMMP2-3	GTCTGGATGCAGCGGAAACA**AGG**
sgMMP2-4	AAACATACAAAGGGATTGCC**AGG**
sgMMP2-5	GCTCCGAGGGTCCGCTGGCT**CGG**

sgMMP2-6	GCGACCCCCGGGCGACGCGC**GGG**
sgMMP2-7	ACGCTGTGTGCCCACCGCCG**AGG**
sgMMP2-8	GGGGGTCTTTGGCAAGCTAT**TGG**
sgMMP2-9	TCCCTTTCCAGTAGTTTGCC**AGG**
sgMMP2-10	ACACATCTGGGCAGTTGCTA**AGG**
sgMMP2-11	ACCAATTTGTCCATTAAGCC**AGG**
sgMMP2-12	ACAACATCTGAACCGCGGTT**TGG**
sgMMP2-13	ACAAGGGAGCAGATATCCCC**TGG**
sgMMP2-14	AGCCTCCCCCTCAACCTGTC**CGG**
