# Supplementary material for: A CRISPR-based approach for targeted DNA demethylation
Source: Cell Discov. 2016 May 3;2:16009–. doi: 10.1038/celldisc.2016.9 (PMC4853773; doi:10.1038/celldisc.2016.9)
Supplement: Supplementary Table S3 [file celldisc20169-s4.pdf]

Supplementary Table 3 Percentages of methylated CpG in total CpGs identified using bisulfite-sequencing method (in *RANKL* gene, stable transfecant cell line).

**Group names**	**Left to right: -700 to TSS site (*RANKL* promoter)**
blank	10%, 20%, 40%, 10%, 30%, 50%, 70%, 50%, 40%
-sgRNA	0%, 30%, 30%, 20%, 20%, 60%, 90%, 80%, 80%
R3: dCas9-CD+MS2-CD	0%, 30%, 10%, 10%, 10%, 20%, 60%, 40%, 50%
R8: dCas9-CD+MS2-CD	0%, 0%, 0%, 10%, 10%, 40%, 90%, 70%, 50%
