# Supplementary material for: A CRISPR-based approach for targeted DNA demethylation
Source: Cell Discov. 2016 May 3;2:16009–. doi: 10.1038/celldisc.2016.9 (PMC4853773; doi:10.1038/celldisc.2016.9)
Supplement: Supplementary Table S4 [file celldisc20169-s5.pdf]

Supplementary Table 4 Percentages of methylated CpG in total CpGs identified using bisulfite-sequencing method (in *MAGEB2* gene).

**Group names**	**Left to right: -700 to -300 site (*MAGEB2* promoter)**
blank	18.2%, 81.8%, 18.2%, 27.3%, 54.5%, 54.5%, 20%, 27.3%
-sgRNA	53.8%, 69.2%, 15.4%, 30.8%,61.5%, 46.2%, 38.5%, 53.8%
M1: dCas9-CD+MS2-CD	33.3%, 66.7%, 16.7%, 41.7%, 66.7%, 25%, 33.3%, 16.7%
M3: dCas9-CD+MS2-CD	9.1%,36.4%, 0%, 9.1%, 36.4%, 27.3%, 9.1%, 63.6%
M4: dCas9-CD+MS2-CD	25%, 41.7%, 8.3%, 0%, 41.7%, 33.3%, 16.7%, 33.3%
M7: dCas9-CD+MS2-CD	36.4%, 36.4%, 0%, 0%, 54.5%, 27.3%, 9.1%, 45.5%
