# Supplementary material for: A CRISPR-based approach for targeted DNA demethylation
Source: Cell Discov. 2016 May 3;2:16009–. doi: 10.1038/celldisc.2016.9 (PMC4853773; doi:10.1038/celldisc.2016.9)
Supplement: Supplementary Table S5 [file celldisc20169-s6.pdf]

Supplementary Table 5 Sequences of the primers used in qRT-PCR assay.

Target gene	Forward primer (5'-3')	Reverse primer (5'-3')	Application
GAPDH	ACCACAGTCCATGCCATCAC	ATGACCTTGCCCACAGCCTT	qRT-PCR
RANKL	ACTATTAATGCCACCGACA	AGGGTATGAGAACTTGGGAT	qRT-PCR
MAGEB2	GCTGCGGGTGTTTCATCCA	TGGTTAGAGGATCTTCGCTTGG	qRT-PCR
MMP2	GATACCCCTTTGACGGTAAGGA	CCTTCTCCCAAGGTCCATAGC	qRT-PCR
CHST15	TCGTGTGGACAGTAAGCAGAT	TGTAAGAAGCCATTACCAAGGTC	Off-target analysis
ULK4	GAAATAACCAACTGGGTCCGT	CTGTGCAGAGTTCCACCACT	Off-target analysis
SGPL1	CAGCTAATTGCATGGAGTGTCG	CCTTGACCATAAACTCTCTGGC	Off-target analysis
TRAPPC9	ACGGCTCCACACTGTATGACT	TCCTCGTAGTTGGGGTAGAAAG	Off-target analysis
TACR2	AGCAAGTCTACCTGGCACTCT	GAAACCTGTGGTTGAGACAGC	Off-target analysis
LPCAT3	GGAGCTGAGCCTTAACAAGTT	CAAAGCAAAGGGGTAACCCAG	Off-target analysis
FRMD4A	TGAAACGGGACACTTAAACTGG	AACCACGGGTCCTGACTTTTT	Off-target analysis
IDH2	CCCGTATTATCTGGCAGTTCATC	ATCAGTCTGGTCACGGTTTGG	Off-target analysis
FARS2	CACATCCTTCCTTTGAGATGGAG	CCAGCCGATTCGGTCTTGA	Off-target analysis
KCNQ2	TGCCTGGTACATCGGCTTC	GTAGGTGTCAAAGTGGTCGTTC	Off-target analysis

Ten pairs of primers used for off-target analysis were derived from the PrimerBank [1].

Supplementary References

1 Spandidos A, Wang X, Wang H, *et al. Nucleic Acids Res* 2010; 38:D792-799.
