# Supplementary material for: A CRISPR-based approach for targeted DNA demethylation
Source: Cell Discov. 2016 May 3;2:16009–. doi: 10.1038/celldisc.2016.9 (PMC4853773; doi:10.1038/celldisc.2016.9)
Supplement: Supplementary Table S6 [file celldisc20169-s7.pdf]

Supplementary Table 6 Sequences of primers used in the bisulfite DNA sequencing.

Target gene	Forward primer (5'-3')	Reverse primer (5'-3')
RANKL-Fragment 1	ATAGAGGTTTTTAAAAAGTTTTAGTAAGGT	ATAATCTCTAAAAACCCTTCCTATCC
RANKL-Fragment 2	ATTTTTTAAATTTTAAGGAGGAAAT	AAAACCAATCAACCCCAAAC
MAGEB2	GGTTTTAGGTTAAAGAGATAAAATTTA	TAAACCTCACAAACCTAACAACAAC
