# Supplementary material for: A CRISPR-based approach for targeted DNA demethylation
Source: Cell Discov. 2016 May 3;2:16009–. doi: 10.1038/celldisc.2016.9 (PMC4853773; doi:10.1038/celldisc.2016.9)
Supplement: Supplementary Methods [file celldisc20169-s8.pdf]

Supplementary Materials I The full amino acid sequence of dCas9-Tet1 CD.

MYPYDVPDYASPKKKRKVEASDKKYSIGLAIGTNSVGWAVITDEYKVPSKKFKVLGNTDRHSIKKNLIG
ALLFDSGETAEATRLKRTARRRYTRRKNRICYLQEIFSNEMAKVDDSFFHRLEESFLVEEDKKHERHPI
FGNIVDEVAYHEKYPTIYHLRKKLVDSTDKADLRLIYLALAHMIKFRGHFLIEGDLNPDNSDVDKLFIQ
LVQTYNQLFEENPINASGVDAKAILSARLSKSRRLENLIAQLPGEKKNGLFGNLIALSLGLTPNFKSNF
DLAEDAKLQLSKDTYDDDLDNLLAQIGDQYADLFLAAKNLSDAILLSDILRVNTEITKAPLSASMIKRY
DEHHQDLTLLKALVRQQLPEKYKEIFFDQSKNGYAGYIDGGASQEEFYKFIKPILEKMDGTEELLVKLN
REDLLRKQRTFDNGSIPHQIHLGELHAILRRQEDFYPFLKDNREKIEKILTFRIPYYVGPLARGNSRFA
WMTRKSEETITPWNFEEVVDKGASAQSFIERMTNFDKNLPNEKVLPKHSLLYEYFTVYNELTKVKYVTE
GMRKPAFLSGEQKKAIVDLLFKTNRKVTVKQLKEDYFKKIECFDSVEISGVEDRFNASLGTYHDLLKII
KDKDFLDNEENEDILEDIVLTLTLFEDREMIEERLKTYAHLFDDKVMKQLKRRRYTGWGRLSRKLINGI
RDKQSGKTILDFLKSDGFANRNFMQLIHDDSLTFKEDIQKAQVSGQGDSLHEHIANLAGSPAIKKGILQ
TVKVVDELVKVMGRHKPENIVIEMARENQTTQKGQKNSRERMKRIEEGIKELGSQILKEHPVENTQLQN
EKLYLYYLQNGRDMYVDQELDINRLSDYDVDAIVPQSFLKDDSIDNKVLTRSDKNRGKSDNVPSEEVVK
KMKNYWRQLLNAKLITQRKFDNLTKAERGGLSELDKAGFIKRQLVETRQITKHVAQILDSRMNTKYDEN
DKLIREVKVITLKSKLVSDFRKDFQFYKVREINNYHHAHDAYLNAVVGTALIKKYPKLESEFVYGDYKV
YDVRKMIAKSEQEIGKATAKYFFYSNIMNFFKTEITLANGEIRKRPLIETNGETGEIVWDKGRDFATVR
KVLSMPQVNIVKKTEVQTGGFSKESILPKRNSDKLIARKKDWDPKKYGGFDSPTVAYSVLVVAKVEKGK
SKKLKSVKELLGITIMERSSFEKNPIDFLEAKGYKEVKKDLIIKLPKYSLFELENGRKRMLASAGELQK
GNELALPSKYVNFLYLASHYEKLKGSPEDNEQKQLFVEQHKHYLDEIIEQISEFSKRVILADANLDKVL
SAYNKHRDKPIREQAENIIHLFTLTNLGAPAAFKYFDTTIDRKRYTSTKEVLDATLIHQSITGLYETRI
DLSQLGGDSPKKKRKVEASGGGSGGGSGGGSGEAAPCDCDGGTQKEKGPYYTHLGAGPSVAAVRELMET
RFGQKGKAIRIEKIVFTGKEGKSSQGCPVAKWVIRRSGPEEKLICLVRERVDHHCSTAVIVVLILLWEG
IPRLMADRLYKELTENLRSYSGHPTDRRCTLNKKRTCTCQGIDPKTCGASFSFGCSWSMYFNGCKFGRS
ENPRKFRLAPNYPLHNYYKRITGMSSEGSDVKTGWIIPDRKTLISREEKQLEKNLQELATVLAPLYKQM
APVAYQNQVEYEEVAGDCRLGNEEGRPFSGVTCCMDFCAHSHKDIHNMHNGSTVVCTLIRADGRDTNCP
EDEQLHVLPLYRLADTDEFGSVEGMKAKIKSGAIQVNGPTRKRRLRFTEPVPRCGKRAKMKQNHNKSGS
HNTKSFSSASSTSHLVKDESTDFCPLQASSAETSTCTYSKTASGGFAETSSILHCTMPSGAHSGANAAA
GECTGTVQPAEVAAHPHQSLPTADSPVHAEPLTSPSEQLTSNQSNQQLPLLSNSQKLASCQVEDERHPE
ADEPQHPEDDNLPQLDEFWSDSEEIYADPSFGGVAIAPIHGSVLIECARKELHATTSLRSPKRGVPFRV
SLVFYQHKSLNKPNHGFDINKIKCKCKKVTKKKPADRECPDVSPEANLSHQIPSRVASTLTRDNVVTVS
PYSLTHVAGPYNRWV

Annotations:

Bold: the sequence for the epitope of Human influenza hemagglutinin (HA);

Blue color: the sequences for the aminal (N-) and carboxyl (C-) nuclear-leading signal (NLS);

Pink color: the sequence of *Streptococcus pyogenes* $Cas9_{D10A,\ H840A}$, dCas9;

Green color: the sequence of the interspaced linker;

Red color: the sequence of the catalytic domain of mouse Tet1 (Tet1-CD).

Supplementary Materials II The full amino acid sequence of MS2-NLS-Tet1 CD.

MASNFTQFVLVDNGGTGDVTVAPSNFANGVAEWISSNSRSQAYKVTCSVRQSSAQKRKYTIKVEVPKVA
TQTVGGVELPVAAWRSYLNMELTIPIFATNSDCELIVKAMQGLLKDGNPIPSAIAANSGIYSAGGGGSG
GGGSGGGG**SPKKKRKVEAS**GGGSGGGSGGGSGEAAPCDCDGGTQKEKGPYYTHLGAGPSVAAVRELMET
RFGQKGKAIRIEKIVFTGKEGKSSQGCPVAKWVIRRSGPEEKLICLVRERVDHHCSTAVIVVLILLWEG
IPRLMADRLYKELTENLRSYSGHPTDRRCTLNKKRTCTCQGIDPKTCGASFSFGCSWSMYFNGCKFGRS
ENPRKFRLAPNYPLHNYYKRITGMSSEGSDVKTGWIIPDRKTLISREEKQLEKNLQELATVLAPLYKQM
APVAYQNQVEYEEVAGDCRLGNEEGRPFSGVTCCMDFCAHSHKDIHNMHNGSTVVCTLIRADGRDTNCP
EDEQLHVLPLYRLADTDEFGSVEGMKAKIKSGAIQVNGPTRKRRLRFTEPVPRCGKRAKMKQNHNKSGS
HNTKSFSSASSTSHLVKDESTDFCPLQASSAETSTCTYSKTASGGFAETSSILHCTMPSGAHSGANAAA
GECTGTVQPAEVAAHPHQSLPTADSPVHAEPLTSPSEQLTSNQSNQQLPLLSNSQKLASCQVEDERHPE
ADEPQHPEDDNLPQLDEFWSDSEEIYADPSFGGVAIAPIHGSVLIECARKELHATTSLRSPKRGVPFRV
SLVFYQHKSLNKPNHGFDINKIKCKCKKVTKKKPADRECPDVSPEANLSHQIPSRVASTLTRDNVVTVS
PYSLTHVAGPYNRWVAAADYKDDDDK

Annotations:

Blue color: the sequence of MS2 coat protein;

Green color: the sequence of the interspaced linker;

Bold: the sequence for the nuclear-leading signal (NLS);

Red color: the sequence of the catalytic domain of mouse Tet1 (Tet1-CD);

Orange color: the sequence of the Flag peptide.
